# Adherence to Mediterranean Diet and Selected Lifestyle Elements among Young Women with Type 1 Diabetes Mellitus from Northeast Poland: A Case-Control COVID-19 Survey

**DOI:** 10.3390/nu13041173

**Published:** 2021-04-02

**Authors:** Monika Grabia, Anna Puścion-Jakubik, Renata Markiewicz-Żukowska, Joanna Bielecka, Anita Mielech, Patryk Nowakowski, Katarzyna Socha

**Affiliations:** Department of Bromatology, Faculty of Pharmacy with the Division of Laboratory Medicine, Medical University of Białystok, Mickiewicza 2D Street, 15-222 Białystok, Poland; monika.grabia@umb.edu.pl (M.G.); anna.puscion-jakubik@umb.edu.pl (A.P.-J.); joanna.bielecka@umb.edu.pl (J.B.); anita.mielech@umb.edu.pl (A.M.); patryk.nowakowski@umb.edu.pl (P.N.); katarzyna.socha@umb.edu.pl (K.S.)

**Keywords:** diabetes mellitus, Mediterranean diet, COVID-19, dietary pattern, metabolic disease, women, nutritional habits, health behaviors, lifestyle, obesity

## Abstract

An appropriate balanced diet and dietary patterns are important at every stage of life, but in the case of young patients with type 1 diabetes mellitus (T1DM), it is especially crucial during the COVID-19 pandemic. The aim of the study was to assess health and nutritional behaviors, mainly adherence to the Mediterranean diet (MD), during the second wave of the COVID-19 pandemic in Poland among women with T1DM, and to compare them with a healthy population. This survey (based on a questionnaire) was conducted in December 2020 and included 219 young women, healthy (*n* = 106) and with T1DM (*n* = 113), from northeast Poland. Over 30% of the study group admitted that they did not engage in any physical activity. A large proportion declared that their screen time was 5–7 h a day (48% in control and 40% in T1DM group). High intakes of sweet-beverages, sweets and red meat, but also low intakes of olive oil, fish and nuts were observed. The vast majority of participants (60% vs. 71%) were moderately adherent to the Mediterranean Diet Adherence Screener (MEDAS). The study demonstrated that despite the similarity between the behaviors of healthy people and those with T1DM, negative health and nutritional practices, such as low physical activity, long screen time, medium and high levels of stress and inappropriate eating habits were observed.

## 1. Introduction

Type 1 diabetes mellitus (T1DM) is an insulin-dependent, multifactorial autoimmune disease, which results in degradation of the beta cells of the islets of Langerhans, which causes impaired insulin production and secretion. The treatment method consists of functional intensive insulin therapy delivered by multiple daily injections (MDIs) using an insulin pen, or a device called personal insulin pump, enabling continuous subcutaneous insulin infusion (CSII), which better mimics the physiological rhythm of insulin secretion [1].

COVID-19 (coronavirus disease 2019) is an acute infectious respiratory disease caused by the SARS-CoV-2 virus (severe acute respiratory syndrome coronavirus). It was first recognized and described in November 2019 in central China (Hubei province). It is considered that the origin of this virus was a seafood market where other animals such as snakes, frogs and bats were also sold. The genome of SARS-CoV-2 is known to be similar to the bat coronavirus and one unrecognized coronavirus, probably the pangolin coronavirus [2].

The Mediterranean diet (MD) is a dietary pattern, the benefits of which are supported by a large body of scientific evidence that highlights the potential health benefits of adherence. Nowadays, the MD pattern should be considered not only from a nutritional perspective but also in the light of environmental, economic as well as sociocultural factors. MD is related to a lower risk of developing several chronic diseases, such as type 2 diabetes mellitus (T2DM), heart disease and cancer [3,4]. MD has also been shown to improve cognitive functions [5]. The recently updated MD model highlights the need for a sustainable approach to this diet, with special emphasis on decreased consumption of meat, high fat dairy products and processed foods, and increased intake of locally grown fruits, vegetables, legumes, olive oil, whole grains and nuts. Fish, poultry and red wine should be consumed in moderate amounts. Moreover, the MD may provide considerable amounts of antioxidants, polyphenols, carotenoids (such as lycopene and β-carotene), as well as dietary fiber [3,4,6,7]. Also, because of the high consumption of olive oil, especially extra virgin and nuts, it is rich in monounsaturated and polyunsaturated fatty acids. Many studies have linked their high consumption with an improvement in insulin sensitivity, blood lipid profile, and a reduction in systolic and diastolic blood pressure levels, in line with the standards of medical care established by the American Diabetes Association [7,8,9].

The aim of the study was to assess health and nutritional behaviors, mainly adherence to MD, during the second wave of the COVID-19 pandemic in Poland among women with T1DM, and to compare them with a healthy population. It was undertaken due to the fact that a number of studies have shown a beneficial effect of the MD in people with diabetes mellitus (DM). This is of particular importance in times of the COVID-19 pandemic. Our research is designed to identify the problem, the solution to which may be inclusion of preventive and educational programs aimed at rectifying possible unhealthy habits. Studies assessing health habits (mainly concerned glycemic management) during the COVID-19 pandemic in healthy people; there are few studies among people with DM, and even fewer among those with T1DM.

## 2. Materials and Methods

### 2.1. Participants

This case-control survey was conducted among 219 young Polish women. The study group consisted of 113 persons with T1DM (52% used MDIs and 48% used CSII) and the control group contained 106 healthy individuals. The median ages in T1DM and healthy groups were 22 and 25 years, respectively. The online survey was carried out in December 2020, during the peak of the second wave of the COVID-19 pandemic, through private groups on social media platforms. The main criterion for inclusion in the study group was young age (between 16 and 35 years) and residence in Warmian-Masurian or Podlaskie Voivodeships. Responses from participants residing abroad, a different type of DM than the type 1 and people who had ever tested positive for the new coronavirus have been rejected. At the same time, a survey was conducted among healthy volunteers who expressed their willingness to participate in our study. Each person was informed that the completed questionnaire was anonymous and confidential. The questionnaire could be completed only once and it was possible to withdraw from the survey at any given moment, then the answers were not saved. By completing and sending the questionnaire, respondents confirmed consent to participate in the study. No personal data were required. The study had obtained the consent of the Bioethical Commission of the Medical University of Bialystok No. R-I-002/587/2019.

### 2.2. Questionnaire

The initial part of the questionnaire included questions that allowed for a reliable selection of study participants, dividing them into groups. The questions concerned the existing diseases, duration of the T1DM and type of treatment, sex, age, place of residence. The body weight and height (self-reported) results were used to calculate the body mass index (BMI), which reflected the general nutritional status of the patient. It was calculated as: weight in kg divided by height in meters squared. In children and adolescents under 18 years of age, it is interpreted according to national standards, and the limits of underweight, overweight and obesity are defined as the 10th, 85th and 97th centiles, respectively [10]. For adults, the values established by the World Health Organization were applied: a person whose BMI is below 18.5 kg/m^2^ is considered underweight, the normal value is 18.5–24.9 kg/m^2^, whereas in overweight and obese persons the values are 25.0–29.9 kg/m^2^ and over 30.0 kg/m^2^, respectively [11]. The results of glycated hemoglobin (HbA1c) from the last 3 months (self-reported) were obtained in a laboratory at the request of the attending physician.

The next part of the survey included questions about lifestyle (sleep time, screen time, stress levels), physical activity and eating habits, including the Mediterranean Diet Adherence Screener (MEDAS), which consists of 14 questions about eating behaviors typical of a MD (Table 1). Each question could earn a point; the maximum number of points to be earned was 14. The responses were to refer to the last month preceding the completion of the questionnaire. Based on the total scores, participants were divided into three levels: low (score 0–5), medium (6–9 points) and high (≥10 points) MD adherence.

The entire questionnaire consisted of questions that had appeared in our previously published study and other authors’ work [12,13]. Questions in foreign languages were translated into Polish and assessed by a native speaker of the Polish language in order to exclude any bias in the translation. The translated questionnaire was tested on a small sample of respondents in order to avoid formal and substantive errors.

### 2.3. Statistical Analysis

Statistical analysis of the results was performed using Statistica software (TIBCO Software Inc., Palo Alto, CA, USA). The Shapiro–Wilk test was applied to check the normal distribution of the variables. According to the test outcomes, Student’s *t*-test (parametric variables), the Mann–Whitney U and Kruskal–Wallis ANOVA tests (non-parametric variables) were used. The Chi-square independence test evaluated the relationships between qualitative features. Before the survey, a required minimum sample size was estimated. It was useful for calculating the total participants of our study with a specified confidence interval (95%) and a maximum bias (10%). Values at *p* < 0.05 were considered statistically significant. The supplementary material contains additional characteristics of the most significant results divided according to variables (place of residence, age group).

## 3. Results

The characteristics of the groups are summarized in Table 2. The distribution of study participants according to insulin therapy was almost equal (52% MDIs vs. 48% CSII users). The majority of patients with T1DM had well-controlled diabetes (glycated hemoglobin —7%). Most of the study respondents (60%) had a normal BMI, 25% and 9%, respectively, were overweight or obese, while 6% were underweight. There were statistically significant differences in body weight and BMI between the healthy and the T1DM groups. People with T1DM had excess body weight and higher BMI more often than healthy persons. The same trend was noticed when the groups were distinguished according to the insulin therapy used. Additionally, in those using pens both parameters were higher compared to users of insulin pumps and healthy persons.

There was a significant dependence (*p* < 0.01) of the frequency of physical activity between the main groups (T1DM vs. healthy). The frequency of physical activity did not affect the type of insulin therapy used—31% of the study group admitted that they did not engage in any physical activity, 38% (39% of CSII and 37% of MDIs users) exercised once or twice a week, 25% (22% and 27%, respectively) exercised three to four times a week, while 6% (7% and 5%, respectively) more than five times a week. Comparing these results to the control group, it was respectively: 15%, 37%, 35% and 13%.

**Figure 1:**
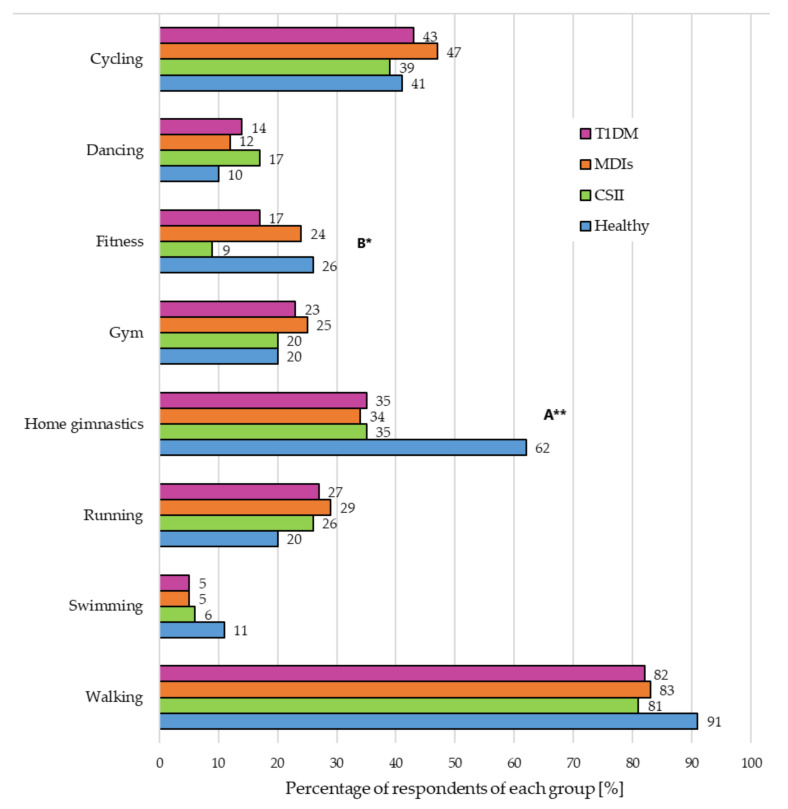
Type of physical activity chosen by study participants during the second wave of the COVID-19 pandemic. Abbreviations: continuous subcutaneous insulin infusion (CSII), multiple daily injections (MDIs), type 1 diabetes mellitus (T1DM). Statistically significant dependence between variables: ^A^ T1DM vs. Healthy, ^B^ CSI vs. MDIs (the Chi-square test), * *p* < 0.05 and ** *p* < 0.001.

Figure 1 shows the type of physical activity chosen by all the study participants. At that time, the most popular pursuits, in both the healthy and TD1DM groups, were walking (over 80% and 40%, respectively) and home gymnastics (62% in control vs. 35% in T1DM group, *p* < 0.001).

Most respondents devoted 5–8 h per day to sleep: 73% of healthy and 46% T1DM persons (a similar percentage was found in both groups on insulin therapy). Over 8 h of sleep was declared by 23% and 46%, respectively (Table 3).

Almost one-third of the respondents in both groups replied that they spent 2–4 h a day in front of a computer or TV. However, in most cases the declared screen time was 5–7 h a day (48% in control and 40% in T1DM group) (Table 3).

Also, there was a characteristic variation in the number of meals for the T1DM group (Table 3). Statistically significantly (*p* < 0.001), people from this group ate more frequently (41% and 54% ate more than five meals or three to four meals a day, while in the group of healthy people it was 20% and 66%, respectively).

**Figure 2:**
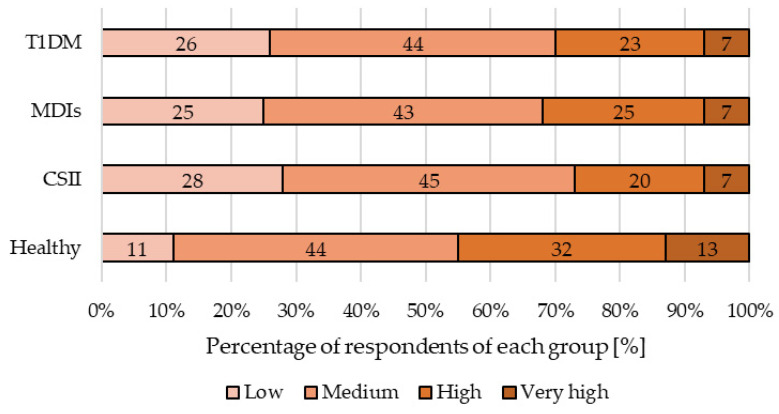
Stress level distribution of study cohort during the second wave of the COVID-19 pandemic. Abbreviations: continuous subcutaneous insulin infusion (CSII), multiple daily injections (MDIs), type 1 diabetes mellitus (T1DM). Statistically significant (*p* < 0.001) dependence between T1DM and healthy (the Chi-square test).

Figure 2 shows stress level percentage distribution in the study cohort during the second wave of COVID-19. The vast majority (44%) declared that they experienced medium stress. Slightly fewer (32% of healthy people and 23% of diabetics) said they still felt highly stressed.

**Figure 3:**
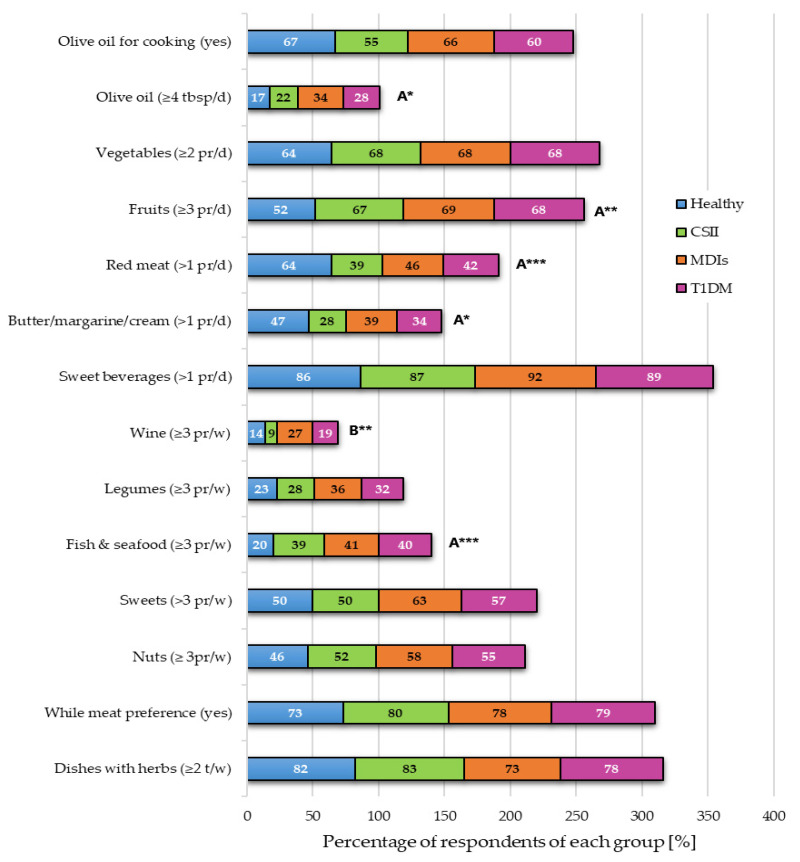
Percentage of respondents consuming certain portion sizes of product groups characteristic of the Mediterranean diet. Values are expressed as percentage of respondents (%). Abbreviations: continuous subcutaneous insulin infusion (CSII), multiple daily injections (MDIs), Mediterranean Diet Adherence Screener (MEDAS), Mediterranean Diet (MD), number of respondents (n), daily (d), weekly (w), portion (pr), tablespoon (tbsp), times (t), type 1 diabetes mellitus (T1DM). Statistically significant dependence between variables: ^A^ T1DM vs. Healthy, ^B^ CSII vs. MDIs (the Chi-square test), * *p* < 0.05, ** *p* < 0.01, *** *p* < 0.001. The size of portion: vegetables 200 g, sweet or beverages 200 mL, meat and fish 100–150 g, legumes 150 g, wine 125 mL, fruits 100 g, nuts 10 g, butter/margarine/cream 12 g.

**Figure 4:**
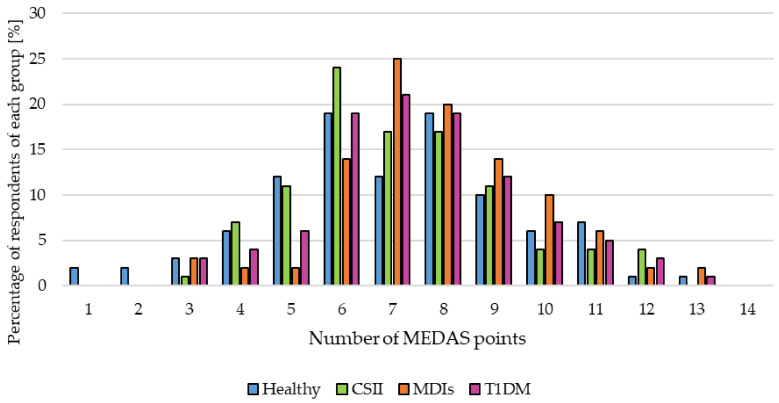
Adherence to the Mediterranean diet in the study cohort. Abbreviations: continuous subcutaneous insulin infusion (CSII), multiple daily injections (MDIs), Mediterranean Diet Adherence Screener (MEDAS), type 1 diabetes mellitus (T1DM).

The respondents were asked whether they consumed a specific number of servings of a given product or group of products characteristic of the MD according to the MEDAS. Figure 3 presents the percentage of people who declared that they consumed this number of portions of a given food. Statistically significant differences between the responses in the main groups (healthy vs. T1DM) were observed for the servings of vegetables, olive oil, fruits, meat, butter/margarine/cream and fish/seafood consumed. There was also a significant relationship between subgroups using different types of insulin therapy (CSII vs. MDIs) as regards the amount of wine consumed (Figure 3). The vast majority were moderately adherent to MEDAS—60% of healthy and 71% of diabetics (Figure 4).

It was observed that diabetic women in the group with high adherence to MEDAS, compared to women with low adherence to MEDAS, more often slept for more than 8 h (50% vs. 40%), spent less time in front of a TV or computer (≥5 h of screen time: 49% vs. 87%) and consumed ≥5 meals a day (44% vs. 27%) (Table 4).

The above results (frequency of physical activity, number of meals, screen and sleep time and stress level) were divided into variables (place of residence, age group) and included in Supplementary Tables S1 and S2.

## 4. Discussion

A properly balanced diet and appropriate dietary patterns are important at every stage of life, but in the case of young patients with T1DM, it is especially crucial since it can prevent or delay the symptoms of many diabetes-related conditions.

The survey was conducted in December 2020, and respondents were asked provide information regarding the previous month. The number of COVID-19 cases recorded in Poland on 1 November was 17,717, and on 23 December—12,358, which was the second peak of the pandemic [14].

Our study showed a high percentage of patients with T1DM who were overweight (32%) or obese (13%). Factors such as increased body weight, low physical activity, long screen time and exposure to stressful situations may lead to diabetic complications. Adherence to the MD has a crucial role in reducing the risk of health consequences.

The restrictions introduced due to the COVID-19 crisis affect various aspects of life. For instance, our research on a group of diabetics, assessing the health consequences of the first wave of the pandemic, showed that the body weight of 31% of respondents had increased by less than 5 kg, while in 11% of the cases—by more than 5 kg [12]. Another study conducted in Poland showed that during the first lockdown, 48.8% of overweight and 55.3% of obese people declared that they ate more, 55.3% and 61.7%, respectively, indicated that they ate more snacks, while 63.3% and 62.6%, respectively, said they cooked more [15]. The research conducted in this study, concerning the period of the second wave, showed BMI above the norm in as many as 45% of respondents with T1DM, which indicates a disturbing trend caused by restrictions on, for example, access to gyms. Research conducted among healthy population, during the first rise in COVID-19 incidence, also showed significant differences in the number of meals consumed. It was shown that during isolation there was an 11.2% increase in the percentage of people who ate five or more meals (from 19.9 to 31.1%) [16]. Being overweight or obese is an increasingly frequent risk factor among people with T1DM, not only in Poland, but also all over the world. It has been demonstrated that in Australia as many as 33% of adolescents under the age of 16 are overweight or obese, and among persons over 18 years of age: 38.3% and 17.2%, respectively [17,18]. Data from Sweden also indicate a large percentage of people over 18 years of age with excess body weight (35.1% of overweight people and 8.9% of obese people) [19]. Our study also showed significant differences in the number of meals consumed by healthy women and those with T1DM. Consumption of five or more meals was declared by 20% and 41%, respectively (*p* < 0.001). At its onset, the pandemic enforced certain social behaviors, such as excessive buying of food and hygiene products for the purpose of creating stocks. The resultant large amounts of products stored at home could be associated with excessive calorie consumption—it has been proven that the number of meals eaten at home increased by 38%. Stressful factors can trigger negative eating behavior, such as snacking between meals, leading to increased caloric value of the diet, and thus obesity [20].

Physical activity is another important element in the prevention of obesity and diabetes complications. Our previous study revealed that during the first wave of the COVID-19 pandemic, the percentage of respondents exercising one to two times a week had increased from 36% (before the pandemic) to 41%. On the other hand, the percentage of people exercising more often had decreased: three to four times a week—from 31% to 19%, more than five times a week—from 12% to 6%. The most common activities were walking and cycling [12]. Our current study found that walking was the physical activity that both people with T1DM (82%) and healthy ones (91%) chose most frequently. Patients with DM also chose cycling (43%) and exercising at home (35%). Regular physical activity improves, among others, sleep quality. There have been reports that during the lockdown period, physical activity, because of its numerous benefits, should be promoted in the same way as other public health related behaviors (including disinfection and distancing). Exercise can be a way to improve both physical and mental health [20,21].

Sleep duration was another factor that was analyzed. We showed that 46% of our respondents slept for more than 8 h—the differences between T1DM and healthy people were statistically significant (*p* < 0.001). Reduction of sleep time has been revealed to play a significant role in the pathogenesis of many chronic diseases. People have more flexibility as regards their sleep hours when they spend more time at home. Usually they fall asleep later and the quality of their sleep is worse: an increase in nocturnal awakenings is observed even when the length of nighttime rest is adequate. Sleep disorders may adversely affect homeostasis, consequently leading to disorders of mood, impaired well-being, worse eating habits, loss of motivation to take up physical activity, eventually resulting in hormonal disorders in obesity and DM [20,22,23,24].

As regards screen time, we have shown significant differences (*p* < 0.01) between patients with CSII and MDIs. It is a concern that as many as 40% of the diabetics involved in our study spent 5 to 7 h in front of a computer or TV, and 24%—8 h or more.

Stress is another factor that may exacerbate the course of many diseases, including T1DM, and trigger the development of long-term complications. The timing of the pandemic resulted in different patterns of coping with stress. Our previous research aiming to assess changes in social behavior among the DM population found that prior to the pandemic, none of the respondents had described their stress levels as ‘very high’. At the beginning of the pandemic, the percentage of people who claimed to be highly stressed was around 32%, while during the study, 4% of respondents rated their stress levels as ‘very high’ and 17% as ‘high’ [12]. Our current results have revealed a tendency towards better control of negative emotions and greater capacity to learn to function in a changed reality. The highest percentage of people assessing their stress level as ‘very high’ was found among healthy people (13%), while among all diabetics, both in the CSII and MDIs groups, the figure was 7%. This may be due to the fact that having been exposed to stress for an extended period of time, they now perceive the new threats differently and are better equipped to face them.

None of the subjects included in our study received the maximum number of points on the MEDAS scale. The most frequent scores were the medium values: from six to nine. The highest percentage of people with MDIs obtained seven points (25%), while the highest percentage of patients with CSII: six points (24%). In the healthy group, 19% of respondents obtained six and eight points each, which proves the need for educational activities that must be carried out in the field of pro-health prophylaxis of patients with T1DM, but also among healthy people.

Metabolic syndrome (MetS) can be another consequence of an improper lifestyle, including inappropriate diet. The impact of cardiovascular disease (CVD) risk factors in adolescents with T1DM is not completely explained. Mayer-Davis et al. conducted a study on a group of 1198 diabetic patients at an average age of 14.83 ± 3.13 years. They showed that CVD risk factors were increased: blood pressure (incidence: 27%), obesity (21%) and high lipid level (18%). The authors concluded that there was little evidence that only a single factor underlay the pattern of CVD risk factors in adolescents with DM [25].

Vidal-Peracho et al. conducted a study to assess compliance with the MD among the inhabitants of Spain—also women with T1DM in the older age group (44.13 ± 12.0 years). The authors, similarly to our study, showed that the average index of the MD among those patients was medium (69%). Interestingly, among the subjects who strictly complied with the recommendations, women constituted a significantly lower percentage than men (22.4% vs. 30.2%). The smallest percentage of women (around 10%) did not follow the recommendation to drink more than seven servings of wine, while the largest proportion complied with the recommendation to use olive oil (around 90%) [26].

The specific components that should be present in the menu of patients following the MD are characterized by multidirectional prophylactic properties and have a positive effect on the parameters of the MetS.

In our study, 46% of healthy people, 52% of CSII and 58% of MDIs patients consumed more than three servings of nuts per week. Although for the majority of diabetic patients oil was the main fat (55% of CSII and 66% of MDIs users), only 22% and 34% of the respondents declared daily consumption of more than four tablespoons. It is worth emphasizing the statistically significant difference (17% vs. 28%, *p* < 0.01) in the frequency of consumption of olive oil between healthy people and those with T1DM. Studies by Grando-Casas et al. showed a positive tendency: patients with T1DM consumed significantly more fatty fish (36.2 vs. 29.2, *p* = 0.009) and nuts (14.7 vs. 9.0, *p* = 0.011) than healthy people [27]. The literature emphasizes the synergistic anti-inflammatory effect of nuts and olive oil, which helps to reduce the health consequences of diabetes. The consumption of fatty acids, including eicosapentaenoic acid (EPA) and docosahexaenoic acid (DHA), contributes to the reduction of inflammation and has cardioprotective action [28].

The main sources of dietary fiber in the MD are: whole grains, vegetables, fruits and nuts. In our study, consumption of three or more servings of legumes per week was reported by 28% of patients with CSII and 36% of patients with MDIs. Vegetables were consumed twice a day or more often by 68% of patients with CSII and MDIs, while fruit was eaten three or more times a day by a similar number of people: 67% of CSII and 69% of MDIs. In patients with T1DM, adherence to the guidelines of the MD has a beneficial effect on the intestinal microflora. This is an important mechanism because T1DM is an autoimmune disease. Proper microflora decreases the permeability of the intestines and modulates the immune system, whereas low consumption of fiber is related to the development of inflammatory diseases [29].

Products recommended by the MD, such as fruits (e.g., berries) and vegetables, are rich in polyphenolic compounds. Their supporting role is especially emphasized in the context of chronic diseases. Cocoa flavan-3-oils are associated with a reduction in the risk of insulin resistance, systemic inflammation, and DM, as well as improved lipid levels, endothelial blood flow, and blood pressure control. Resveratrol and quercetin also play an important part in cardiometabolic protection. Polyphenols can influence the composition of the intestinal microflora and can also be metabolized to bioactive compounds by intestinal bacteria [30]. The mechanism of action of polyphenols is based on inhibition of intestinal glucose absorption by sodium-dependent glucose transporter 1 (SGLT1), increasing insulin secretion and insulin-dependent glucose uptake, and decreasing hepatic glucose production [31]. There are also reports in the literature that it might be possible to treat DM with polyphenols influencing the AMP-activated protein kinase pathway [32].

We have observed high figures as regards to consumption of sweet beverages of more than one serving per day in 87% of diabetics with CSII and in 92% of diabetics with MDIs. Consuming less than three servings of sweets in a week was reported by 50% of the members of the CSII group and 47% of patients with MDIs. Granado-Casas et al. assessed the compliance with the MD recommendations among patients with T1DM and healthy subjects and showed that diabetic patients consumed significantly fewer sweets (17.4 g vs. 38.5 g, *p* < 0.001) [27]. Patients with insulin resistance and DM are aware of the health consequences of consuming sweet snacks, i.e., excessive body weight and increased insulin resistance, leading to glucotoxicity and accelerated apoptosis of B lymphocytes. Subsequently, immunogenicity is increased, and then symptomatic diabetes develops. In insulin resistance, there is an overload of β cells, which accelerates apoptosis and immune damage [33,34,35,36]. It has been shown that obesity and deteriorated self-management that occur in patients with T1DM are significantly associated with the risk of hospitalization for heart failure, as well as retinopathies and macrovascular diseases [17,19,37]. Obese people have three times higher incidence of low-cholesterol high-density lipoprotein (HDL-C) hypolipidemia and four times higher incidence of hypertension compared to normal body weight [38].

The recommendations of the MD include drinking good-quality red wine in moderate amounts. Valerio et al. assessed the relationship between alcohol consumption as well as cigarette smoking and CVD risk factors in adolescents with T1DM. It was shown that 10% of respondents consumed alcohol and smoked cigarettes. Adolescents who drank alcohol and smoked had higher triglyceride levels compared to those who did not (86.9 vs. 63.9 mg/dL, *p* = 0.01) and lower compliance to MD (6 vs. 7) [39].

Other authors who studied adherence to the MD recommendations among people with T1DM also assessed anthropometric and biochemical parameters. Fortin et al. conducted a 6-month nutritional intervention based on the use of an MD and a low-fat diet in patients with T1DM. Changes in anthropometric parameters were observed in the MD group: waist circumference decreased by 1.5 cm and BMI by 0.7 kg/m^2^. There was also a reduction in systolic blood pressure (from 137 ± 20 to 134 ± 17 mmHg), diastolic blood pressure (from 79 ± 9 to 77 ± 10 mmHg), LDL-cholesterol (from 1.92 ± 0.67 to 1.81 ± 0.61 mmol/L) and triglycerides (from 1.14 ± 0.069 to 0.93 ± 0.44 mmol/L), but these differences were not statistically significant. The need for long-term use of the above-mentioned diet is emphasized in order to obtain greater improvement in parameters [40].

The study by Zhong et al. was designed to determine the relationship between adherence to the MD and glycemic control in adolescents (<20 years of age) with T1DM. It should be stressed that at the beginning of the study only 3% of the 793 participants obtained a high result (score ≥ 8) regarding the compliance with the MD, 46%—a medium (score from 4 to 7) result, and 51.5%—a low result. People with a high index of the quality of the MD had significantly lower total cholesterol compared to those with a low and medium index (143.6 vs. 161.6 and 157.7 mg/dL) and LDL cholesterol (77.1 vs. 95.5 and 91.8 mg/dL) [41].

One of the consequences of DM is cognitive impairment, especially in terms of verbal memory. Kössler et al. assessed the impact of adherence to the MD in patients with T1DM and T2DM. A beneficial effect on cognitive functions was found in patients with T2DM only, which requires further research [42].

Our study has several limitations. Being retrospective, like many studies from the COVID-19 pandemic period, we left it to the patients to estimate the portions consumed, and they may have been biased. Our survey was conducted only among the inhabitants of northeast Poland; therefore, subsequent studies should be based on a broader population sample from other regions of the country with a large number of cases. The study was conducted among women because they are willing to take part in various types of research far more often than men. Moreover, in Poland the percentage of young women with T1DM is much higher than that of men [43]. However, this can be considered an advantage of this study because we had a group that was homogeneous in terms of age and gender (only women) and resided in neighboring provinces, which provided an overview of a larger region—northeast Poland.

## 5. Conclusions

Despite the similarities between the behaviors of healthy people and those with T1DM, undesirable nutritional and health habits were observed during the second wave of the COVID-19 pandemic in both groups. The nutritional patterns of those groups were moderately consistent with the MD. Therefore, it is advisable to promote nutritional and health education in order to increase the awareness of the issue among healthy individuals and those with chronic diseases such as DM. The impact of the aforementioned interventions, with particular emphasis on the above results, would have a positive impact on behavior change, but also on improving treatment results. In the times of the COVID-19 pandemic, new guidelines should be developed based on, for example, the MD pyramid combined with local products.

## Figures and Tables

**Table 1:** Interpretation of Mediterranean Diet Adherence Screener.

Question	Answer: Yes [Points]	Answer: No [Points]
1. Is olive oil the major dietary fat in your diet?	1	0
2. Do you consume at least 4 tablespoons of vegetable oil every day?	1	0
3. Do you eat at least 2 servings (about 400 g) of vegetables every day?	1	0
4. Do you eat at least 3 servings (about 240 g) of fruit every day?	1	0
5. Do you eat less than 1 serving of red meat/other meat products every day?	1	0
6. Do you eat less than 1 serving of butter, margarine or cream every day?	1	0
7. Do you consume less than 1 serving of sweet or sugar-sweetened fizzy drinks every day?	1	0
8. Do you consume more than 3 glasses (approx. 400 mL) of wine per week per week?	1	0
9. Do you eat at least 3 servings (approx. 450 g) of legume seeds (peas, beans, broad beans, lentils, chickpeas) per week?	1	0
10. Do you eat at least 3 servings (approx. 300 g) of fish or seafood weekly?	1	0
11. Do you consume less than 3 servings of sweets (bought, homemade) weekly?	1	0
12. Do you eat at least 30 g of nuts per week?		
13. Do you choose chicken, turkey or rabbit instead of veal, pork or sausage?	1	0
14. Do you eat pasta, vegetable or rice dishes with garlic, tomatoes, leeks or onions more than twice a week?	1	0
TOTAL	14	0

Category: low (score 0–5), medium (6–9 points) and high (≥10 points) Mediterranean Diet adherence.

**Table 2:** Baseline characteristic of study groups.

Studied Parameters	T1DM			Healthy (*n* = 106)
	Total (*n* = 113)	CSII (*n* = 54)	MDIs (*n* = 59)	
Age (years)	25 (20–29)	21 (18–25)	28 (23–32)	22 (21–23)
Body weight (kg) ^A^**^, B^*^, C^**	71 (61–79)	68 (61–78)	72 (62–80)	60 (56–68)
Height (cm)	170 (165–174)	170 (165–175)	169 (163–174)	168.5 (163–173)
Body mass index (kg/m^2^) ^A^**^, C^**	24.4 (21.6–27.7)	23.6 (21.7–27.0)	25.4 (22.4–28.4)	21.9 (19.6–24.1)
HbA1c (%) ^F^	7.1 (6.5–8.0)	7.0 (6.6–7.8)	7.3 (6.4–8.2)	-
Place of residence				
Village	15%	15%	15%	24%
City (≤150 k inhabitants)	29%	24%	32%	27%
City (150–250 k inhabitants)	27%	30%	25%	8%
City (≥250 k inhabitants)	29%	31%	27%	42%
Duration of disease ^E^**				
Up to 5 years	18%	28%	8%	-
5–10 years	26%	35%	17%	-
More than 10 years	56%	37%	75%	-
Body Mass Index ^D^**				
Underweight	5%	7%	3%	8%
Normal	50%	56%	45%	72%
Overweight	32%	32%	32%	17%
Obesity	13%	5%	20%	3%

Values are expressed as median, lower, and upper quartile (Me (Q1–Q3)) or percentage of respondents (%). Abbreviations: continuous subcutaneous insulin infusion (CSII), multiple daily injections (MDIs), number of respondents (n), type 1 diabetes mellitus (T1DM). ^A^ Statistically significant difference between the medians, T1DM vs. Healthy (the Mann–Whitney U test). Statistically significant difference between the medians: ^B^ CSII vs. MDIs, ^C^ MDIs vs. Healthy (the ANOVA Kruskal–Wallis test). Statistically significant dependence between variables: ^D^ T1DM vs. Healthy, ^E^ CSII vs. MDIs (the Chi-square test). ^F^ Results of the glycated hemoglobin (HbA1c) test were collected from 79% of respondents. * *p* < 0.01 and ** *p* < 0.001.

**Table 3:** Frequency of selected healthy behaviors.

Studied Parameters	T1DM			Healthy (*n* = 106)
	Total (*n* = 113)	CSII (*n* = 54)	MDIs (*n* = 59)	
Sleep length ^A^**				
<5 h	8%	8%	8%	4%
5–8 h	46%	46%	46%	73%
>8 h	46%	46%	46%	23%
Screen time ^B^*				
<2 h	10%	3%	17%	5%
2–4 h	26%	26%	27%	28%
5–7 h	40%	54%	27%	48%
≥8 h	24%	17%	29%	18%
Number of meals ^A^**				
1–2 times/day	5%	8%	3%	14%
3–4 times/day	54%	46%	61%	66%
≥5 times/day	41%	46%	36%	20%

Values are expressed as percentage of respondents (%). Abbreviations: continuous subcutaneous insulin infusion (CSII), multiple daily injections (MDIs), number of respondents (n), type 1 diabetes mellitus (T1DM). Statistically significant dependence between variables: ^A^ T1DM vs. Healthy, ^B^ CSII vs. MDIs (the Chi-square test), * *p* < 0.01 and ** *p* < 0.001.

**Table 4:** Health behaviors depending on the Mediterranean Diet Adherence Screener (MEDAS) score category.

	Low Medas (*n* = 41)				Medium Medas (*n* = 144)				High Medas (*n* = 34)			
Studied Parameters	T1DM			Healthy (*n* = 26)	T1DM			Healthy (*n* = 64)	T1DM			Healthy (*n* = 16)
	Total (*n* = 15)	CSII (*n* = 11)	MDIs (*n* = 4)		Total (*n* = 80)	CSII (*n* = 37)	MDIs (*n* = 43)		Total (*n* = 18)	CSII (*n* = 6)	MDIs (*n* = 12)	
Weekly activity												
No activity	40%	46%	25%	35%	25%	24%	26%	9%	50%	50%	50%	6%
1–2 times/week	33%	27%	50%	31%	40%	46%	35%	41%	42%	17%	42%	31%
3–4 times/week	27%	27%	25%	27%	26%	20%	32%	36%	8%	33%	8%	44%
≥5 times/week	-	-	46%	7%	9%	10%	7%	14%	-	-	-	19%
Sleep length												
<5 h	60%	64%	50%	12%	8%	8%	7%	2%	17%	17%	17%	81%
5–8 h	-	-	-	81%	45%	41%	49%	67%	33%	50%	33%	-
>8 h	40%	36%	50%	7%	47%	51%	44%	31%	50%	33%	50%	19%
Screen time												
<2 h	-	-	-	12%	10%	2%	17%	2%	22%	17%	25%	12.5%
2–4 h	13%	9%	25%	15%	26%	22%	30%	34%	39%	83%	17%	25%
5–7 h	47%	55%	25%	58%	45%	62%	30%	44%	11%	-	17%	50%
≥8 h	40%	36%	50%	15%	19%	14%	23%	20%	28%	-	41%	12.5%
Stress level												
Low	47%	37%	75%	12%	25%	30%	21%	10%	39%	37%	75%	13%
Medium	20%	27%	-	42%	44%	49%	4%	45%	17%	27%	-	42%
High	13%	18%	-	19%	25%	16%	32%	34%	27%	18%	-	18%
Very high	20%	18%	25%	27%	6%	5%	7%	11%	17%	18%	25%	27%
Number of meals												
1–2 times/day	20%	18%	25%	19%	39%	5%	67%	14%	28%	67%	8%	6%
3–4 times/day	53%	55%	50%	54%	19%	41%	-	69%	28%	-	42%	75%
≥5 times/day	27%	27%	25%	27%	42%	54%	33%	17%	44%	33%	50%	19%

Values are expressed as percentage of respondents (%). Abbreviations: continuous subcutaneous insulin infusion (CSII), multiple daily injections (MDIs), Mediterranean Diet Adherence Screener (MEDAS), number of respondents (*n*), type 1 diabetes mellitus (T1DM). Category: low (score 0–5), medium (6–9 points), and high (≥10 points) Mediterranean Diet adherence.

## Data Availability

The datasets generated and analyzed during the current study are available from the corresponding author on reasonable request.

## Supplementary Materials

The following are available online at https://www.mdpi.com/article/10.3390/nu13041173/s1, Table S1. Health behaviors depending on the age group; Table S2. Health behaviors depending on the place of residence.
