# Supplementary material for: Adherence to Mediterranean Diet and Selected Lifestyle Elements among Young Women with Type 1 Diabetes Mellitus from Northeast Poland: A Case-Control COVID-19 Survey
Source: Nutrients. 2021 Apr 2;13(4):1173. doi: 10.3390/nu13041173 (PMC8066783; doi:10.3390/nu13041173)
Supplement: Supplementary file 1 [file nutrients-13-01173-s001.pdf]

nutrients

MDPI

Supplementary Material

Adherence to Mediterranean Diet and Selected Lifestyle Elements among Young Women with Type 1 Diabetes Mellitus from Northeast Poland: A Case-Control COVID-19 Survey

Monika Grabia †, Anna Puścion-Jakubik †, Renata Markiewicz-Żukowska*, Joanna Bielecka, Anita Mielech, Patryk Nowakowski and Katarzyna Socha

[1] Department of Bromatology, Faculty of Pharmacy with the Division of Laboratory Medicine, Medical University of Białystok, Mickiewicza 2D Street, 15-222 Białystok, Poland; bromatos@umb.edu.pl

* Correspondence: renmar@poczta.onet.pl ; Tel.: (+48) 85 748 5469

† These authors contributed equally to this work.

Table S1. Health behaviors depending on the age group.

Studied parameters	Adolescents & Students (16-25 years old) T1DM (*n*=60)	Adolescents & Students (16-25 years old) Healthy (*n*=87)	Adults (26-35 years old) T1DM (*n*=53)	Adults (26-35 years old) Healthy (*n*=19)
Weekly activity				
No activity	28%	16%	34%	10%
1–2 times/week	39%	33%	38%	53%
3-4 times/week	28%	36%	21%	32%
≥5 times/week	5%	15%	7%	5%
Sleep length				
<5 h	5%	5%	11%	0%
5-8 h	47%	72%	45%	74%
> 8 h	48%	23%	43%	26%
Screen time				
<2 h	5%	6%	17%	5%
2-4 h	28%	24%	25%	47%
5-7 h	47%	52%	32%	32%
≥8 h	20%	18%	26%	16%
Stress level				
Low	28%	11%	25%	5%
Medium	47%	45%	40%	42%
High	20%	31%	26%	37%
Very high	5%	13%	9%	16%
Number of meals				
1-2 times/day	7%	14%	4%	16%
3-4 times/day	53%	64%	55%	74%
≥5 times/day	40%	22%	42%	10%
Adherence to MD				
Low MEDAS	16%	29%	10%	5%
Medium MEDAS	77%	56%	64%	79%
High MEDAS	7%	15%	26%	16%

Values are expressed as percentage of respondents (%). Abbreviations: Mediterranean Diet Adherence Screener(MEDAS), number of respondents (n), type 1 diabetes mellitus (T1DM). Category: low (score 0–5), medium (6–9 points), and high (≥ 10 points) Mediterranean Diet adherence.

Table S2. Health behaviors depending on the place of residence.

Studied parameters	Village		City (≤150 k inhabitants)		City (150–250 k inhabitants)		City (≥250 k inhabitants)	
	T1DM (n=17)	Healthy (n=25)	T1DM (n=32)	Healthy (n=29)	T1DM (n=31)	Healthy (n=8)	T1DM (n=33)	Healthy (n=44)
Weekly activity								
No activity	35%	20%	28%	17%	32%	0%	30%	14%
1–2 times/week	29%	32%	44%	41%	39%	25%	37%	39%
3-4 times/week	29%	36%	19%	35%	23%	50%	30%	32%
≥5 times/week	7%	12%	9%	7%	6%	25%	3%	15%
Sleep length								
<5 h	18%	0%	9%	3%	7%	0%	3%	7%
5-8 h	52%	84%	44%	62%	48%	38%	42%	80%
> 8 h	29%	16%	47%	35%	45%	62%	55%	13%
Screen time								
<2 h	35%	4%	9%	7%	10%	25%	0%	7%
2-4 h	18%	24%	28%	28%	23%	50%	33%	25%
5-7 h	29%	52%	41%	48%	44%	25%	40%	47%
≥8 h	18%	20%	22%	17%	23%	0%	27%	21%
Stress level								
Low	29%	8%	19%	3%	26%	25%	34%	14%
Medium	53%	69%	56%	41%	32%	50%	36%	36%
High	18%	32%	19%	21%	26%	25%	27%	41%
Very high	0%	0%	6%	33%	16%	0%	3%	9%
Number of meals								
1-2 times/day	0%	4%	3%	24%	16%	13%	0%	14%
3-4 times/day	53%	76%	53%	62%	42%	74%	67%	61%
≥5 times/day	47%	20%	44%	14%	42%	13%	33%	25%
Adherence to MEDAS								
Low MEDAS	23%	24%	9%	31%	16%	0%	9%	25%
Medium MEDAS	59%	64%	78%	55%	71%	63%	70%	55%
High MEDAS	18%	12%	13%	14%	13%	37%	21%	20%

Values are expressed as percentage of respondents (%). Abbreviations: Mediterranean Diet Adherence Screener(MEDAS), number of respondents (n), type 1 diabetes mellitus (T1DM). Category: low (score 0–5), medium (6–9 points), and high (≥ 10 points) Mediterranean Diet adherence.
